# Changes in expression of *C2cd4c* in pancreatic endocrine cells during pancreatic development

**DOI:** 10.1002/1873-3468.12271

**Published:** 2016-07-14

**Authors:** Hisayoshi Omori, Soichiro Ogaki, Daisuke Sakano, Mutsumi Sato, Kahoko Umeda, Naoki Takeda, Naomi Nakagata, Shoen Kume

**Affiliations:** ^1^ Institute of Molecular Embryology and GeneticsKumamoto UniversityJapan; ^2^ Department of Life Science and TechnologySchool of Life Science and TechnologyTokyo Institute of TechnologyYokohamaJapan; ^3^ Division of PharmacologyNational Institute of Health ScienceKamiyogaSetagaya‐kuTokyoJapan; ^4^ HIGO programKumamoto UniversityJapan; ^5^ Division of Developmental GeneticsInstitute of Resource Development and AnalysisKumamoto UniversityJapan; ^6^ Division of Reproductive EngineeringInstitute of Resource Development and AnalysisKumamoto UniversityJapan

**Keywords:** C2cd4, insulin, pancreas, pancreatic beta cell

## Abstract

C2cd4c, encoded by a gene belonging to the *C2cd4* family, contains a C2 domain conserved across species and is localized to the cytoplasm. To examine the role of C2cd4c in the pancreas, we studied its localization and generated *C2cd4c* knockout (KO) mice. C2cd4c was expressed in pancreatic endocrine progenitors at early embryonic stages. When endocrine cells arise from their precursors, C2cd4c is gradually confined to the insulin‐ and pancreatic polypeptide‐expressing cells of the endocrine. In the adult pancreas, C2cd4c is restricted to the beta cells. *C2cd4c* KO mice showed normal embryonic pancreatic development and adult pancreatic function. Thus, our results suggest that *C2cd4c* is dispensable for pancreatic development.

## Abbreviations

***C2cd4b***, *C2 calcium‐dependent domain‐containing protein 4B*

**DBA**, *Dolichos biflorus* agglutinin

**ES**, embryonic stem

**IPGTT**, intraperitoneal glucose tolerance test

**KI**, knock‐in

**KOMP**, Knock Out Mouse Project

**NBT**, Nitro Blue tetrazolium

**ORF**, open reading frame

The pancreas is composed of acini that produce digestive enzymes for digestion of food, endocrine cells that produce hormones, and duct cells. All these lineages are derived from Pdx1‐expressing multipotent pancreatic progenitor cells 1. The endocrine compartment consists of hormone‐producing endocrine cells, including insulin‐producing beta cells, glucagon‐producing alpha cells, somatostatin‐producing delta cells, and pancreatic polypeptide‐producing PP cells 2, 3. In mice, differentiation of pancreatic endocrine cells from the endocrine progenitor cells occurs approximately at embryonic day 14.5 (E14.5). Multipotent progenitors are known to exist in particular regions of the developing pancreas. Endocrine progenitor cells are located in the ‘trunk region,’ whereas acinar progenitor cells are located in the ‘tip region’ of the developing pancreas 4, 5. During this period, Nkx6.1‐expressing early endocrine progenitor cells differentiate into Ngn3‐expressing endocrine progenitor cells 1, 6, which then give rise to insulin‐expressing beta cells. After birth, pancreatic endocrine cells form the pancreatic islets 7. Deletion of either Nkx6.1 or Ngn3 causes abnormal pancreatic endocrine development 8, 9.

Previously we found that the gene *C2 calcium‐dependent domain‐containing protein 4B* (*C2cd4b*) is expressed in pancreatic β cells, as assessed by analyzing the gene expression profile of pancreatic progenitor cells derived from mouse ES cells. We confirmed the expression of *C2cd4b* in the embryonic pancreas by *in situ* hybridization 4. *C2cd4b* and *C2cd4a* are members of the *C2cd4* family, and the *C2CD4A*‐*C2CD4B* locus has been identified as a risk factor for type 2 diabetes 10. *C2cd4c* is another member of the *C2cd4* family whose expression has not been reported. The *C2cd4c* gene product possesses the C2 domain with the Ca^2+^‐binding motif, which is well conserved across species 11. In contrast, *C2cd4a* and *C2cd4b* gene products do not have the C2 domain. In the present study, we focused on C2cd4c and generated *C2cd4c*/*LacZ* knock‐in (KI) mice to monitor its expression patterns and to investigate its functions.

## Materials and methods

### Reverse transcription and semiquantitative PCR analysis

RNA was extracted from the cells using the RNeasy Micro‐Kit (Qiagen, Heiden, Germany) and then treated with DNase I (Qiagen). Complementary DNA was synthesized from 3 μg of total RNA using ReverTra Ace (Toyobo, Osaka, Japan) and oligo(dT) primers. For the semiquantitative PCR analysis, DOD Dash (Toyobo) was used, and the PCR conditions were as follows: denaturation at 95 °C for 30 s, annealing at 60 °C for 2 s, and extension at 72 °C for 45 s. RT‐PCR products were separated by 5% nondenaturing PAGE, stained with SYBR Green I (Molecular Probes, Waltham, MA, USA), and visualized using kodak 1d software (Eastman Kodak Company, Rochester, NY, USA). All primers used are listed in Table S1.

### Sequence alignments

Sequence alignments of published mouse C2CD4A (accession: gi 253314502, NP_001156615.1), C2CD4B (gi 124486785, NP_001074783.1), C2CD4C (gi 274323057, NP_001162095.1), C2CD4D (gi 209870113, NP_001129589.1), C2CD4C from human (hC2CD4C, gi 152032539, sp Q8TF44.2), *Macaca mulatta* (maC2CD4C, gi 302563773, NP_001181749.1), rat (rC2CD4C, gi 672058048, XP_008763400.1), *Xenopus tropicalis* (xC2CD4C, gi 213983019, NP_001135670.1), and chick (chC2cd4c, gi 971436334, XP_015155493.1) were performed using the NCBI Constraint‐based Multiple Alignment Tool (COBALT) and Conserved Domain Data base (CDD).

### Cell culture

MIN6 and COS‐7 cells were cultured in Dulbecco's modified Eagle's medium (DMEM) (Invitrogen, Tokyo, Japan) supplemented with 10% FBS (Hyclone, Buckinghamshire, UK), 50 units·mL^−1^ of penicillin, and 50 μg·mL^−1^ of streptomycin (PS, Nacalai Tesque, Kyoto, Japan) in 5% CO_2_.

### Overexpression of Flag‐tagged C2cd4c

Flag‐tagged or HA‐tagged *C2cd4c* was introduced into pCDNA3. The primer sequences for the expression vector were FLAG‐C2cd4c‐forward: ATGGACTACAAAGACGATGACGACAA‐ GAGAAAAACCAACATGTGGTTCTT, FLAG‐C2cd4c‐reverse: TCACAGAAAGGGCAACAGGG, C2cd4c‐FLAG forward: TGTGGTTCTT, and C2cd4c‐FLAG reverse: TCACTTGTCGTCATCGTCTTTGTAGTCCAGAAAGGGCAACAGGG. C2cd4c‐HA forward: ATGAGAAAAACCAACATGTGGTTCTTG, C2cd4c‐HA reverse: TCAAGCGTAATCTGGAACATCGTATGGGTACATGGTTAGCAGTAGCAGAGAGCCCA, HA‐C2cd4c forward: ATGTACCCATACGATGTTCCAGATTACGCTAGAAAAACCAACATGTGGTTC and HA‐C2cd4c reverse: TCACAGAAAGGGCAACAGGGA.

The expression vectors were transfected into MIN6 and COS‐7 cells using Fugene HD (Promega, Madison, WI, USA), and analyzed with anti‐FLAG or anti‐HA antibodies. Fluorescent images of the transfected cells were acquired using a confocal microscopy (Leica TCS SP2 AOBS, Wetzlar, Germany).

### *C2cd4c*‐*LacZ*/KI ES cells

Embryonic Stem (ES) cells (JM8A3‐A10) were obtained from the Knock Out Mouse Project (KOMP) Repository 12, 13, 14.

### Southern blotting

Genomic DNA was digested with *Eco*R1 (Toyobo). After digestion, the fragmented genomic DNA was separated by 0.7% agarose gel electrophoresis and then transferred to Hybond N Plus (GE Healthcare UK Ltd., Buckinghamshire, UK) and cross‐linked by UV Stratalinker (Stratagene, Tokyo, Japan). The membranes were then hybridized with digoxigenin‐labeled probes using a DIG Easy Hyb kit (Roche, Basel, Switzerland). The primer sequences for the DNA probes are 5′‐*C2cd4c*‐forward; GCCAGGTCAAGCTGTTCTTC, 5′‐*C2cd4c*‐reverse; CAGTCATGGGCACTCAGCTA, 3′‐*C2cd4c*‐forward; CAGCCATACCTTGGAGTGGT, 3′‐*C2cd4c*‐reverse; TCCAGAGCAAAGTGCATGAG.

### Antibodies

For immunohistochemical analysis, rabbit anti‐Pdx1 (1 : 200, KR059, Lot# TG080814; Trans Genic Inc., Fukuoka, Japan), goat anti‐Ngn3 (1 : 200, PS36, a gift from G. Gu, Vanderbilt University), mouse anti‐Nkx6.1 (1 : 100, F64A6B4; Developmental Studies Hybridoma Bank, Iowa, IA, USA), guinea pig anti‐Insulin (1 : 1000, A0564, Lot#10079943A; Dako Cytomation, Tokyo, Japan), mouse anti‐Glucagon (1 : 1000, G2654, Lot# 12M4084; Sigma‐Aldrich, St. Louis, MO, USA), goat anti‐Somatostatin (1 : 300, sc‐7819; Santa Cruz Biotechnology Inc., Dallas, TX, USA), rabbit anti‐Pancreatic Polypeptide (1 : 300, A0619; Dako Cytomation), rabbit anti‐Amylase (1 : 100, A8273; Sigma‐Aldrich) and mouse anti‐FLAG M2 (1 : 2000, F1804; Sigma‐Aldrich), anti‐HA antibody (1 : 2000, 011‐21911; Wako, Osaka, Japan) were used.

### 6‐chloro‐3‐indolyl‐beta‐d‐galactopyranoside (S‐gal) and SPiDER‐βGal staining

S‐gal and SPiDER‐βGal (Dojin) staining were performed to visualize β‐galactosidase activity 15, 16.

### Measurement of blood glucose levels

Blood glucose levels were measured using the Life Check Sensor (Gunze, Osaka, Japan). For glucose tolerance testing, mice were fasted for 16 h, after which blood glucose levels were measured at indicated time points after intraperitoneal glucose administration at 2 mg per body weight (g) 17.

## Results

### C2cd4c is a C2 domain‐containing protein localized to the cytoplasm

**Figure 1:**
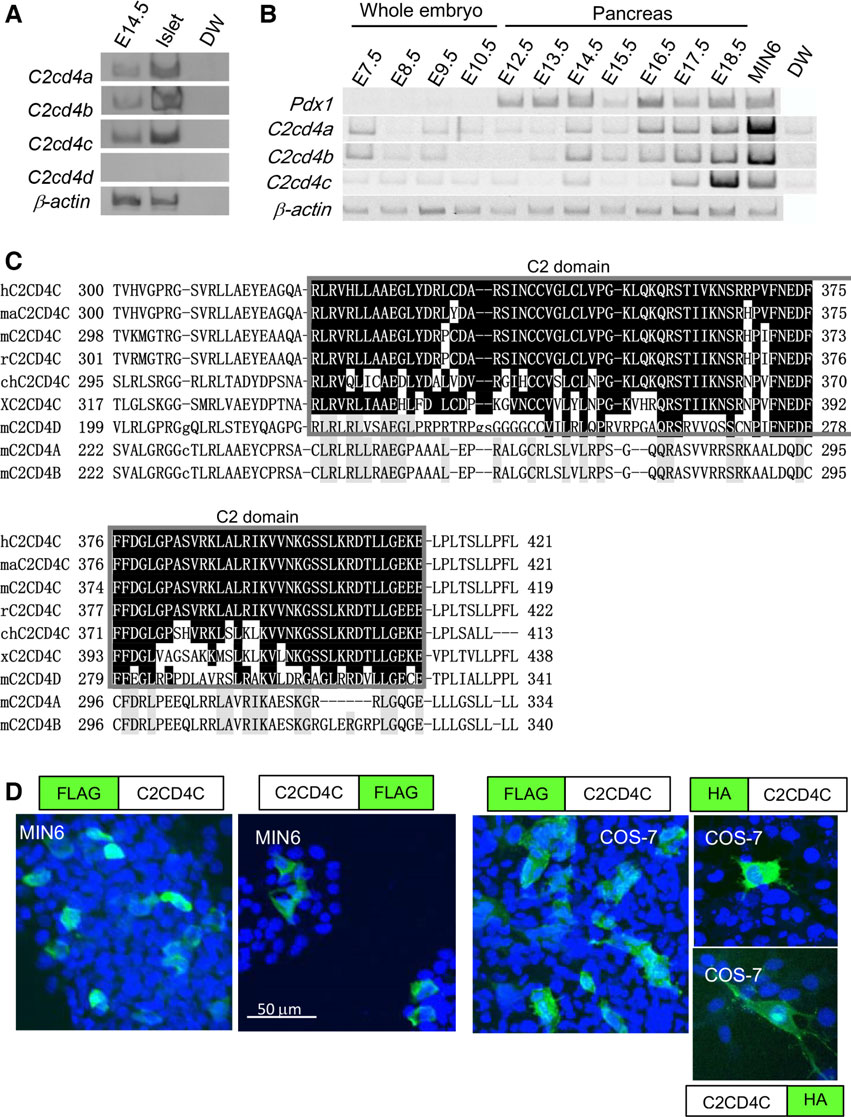
C2cd4c is expressed in embryonic and adult pancreas and is a C2 domain‐containing protein localized to the cytoplasm. (A, B) Semiquantitative RT‐PCR analysis of *C2cd4* family genes in the embryonic pancreas at E14.5 and in adult islets (A), and stage‐dependent expression of *C2cd4a*,* C2cd4b,* and *C2cd4c* in early embryonic stages in the developing pancreas (B). Also shown are β‐actin as loading control and *Pdx1* as a marker for pancreatic cells. MIN6 is used as a further positive control and distilled water (DW) as a negative control. (C) Sequence alignment of C2 domains (highlighted in black, depicted in a gray box) from human, *Macaca mulatto*, mouse, rat, chick, and *Xenopus tropicalis* C2CD4C. Sequence alignment reveals that C2 domains are highly conserved across species. C2CD4A or C2CD4B do not contain a C2 domain. C2CD4D contains a shorter C2 domain compared to C2CD4C. Homologous amino acids in the C2 domain of C2CD4C are highlighted in black. Identical amino acids in C2CD4A, C2CD4B, and C2CD4D, which are not regarded as C2 domain, are highlighted in gray. (D) Intracellular localization of C2CD4C in MIN6 and COS‐7 cells. FLAG‐ or HA‐tagged C2CD4C, with the tag at either the N‐ or the C‐terminal end, were overexpressed in MIN6 and COS‐7 cells. Immunocytochemical analysis using anti‐FLAG or anti‐HA antibodies revealed that C2CD4C is localized in the cytoplasm. Scale bar; 50 μm.

Previously, we identified *C2cd4b* expression in pancreatic beta cells 4. *C2cd4b* is a member of the *C2cd4* family, consisting of *C2cd4a*,* C2cd4b*,* C2cd4c,* and *C2cd4d*. The *C2cd4* family genes, except *C2cd4d*, were expressed in early stages in the embryonic pancreas and in the adult islets (Fig 1A,B). Among the *C2cd4* family genes, C2CD4C and C2CD4D contain the C2 domain, which is well conserved across species (Fig 1C). Of note, C2CD4A and C2CD4B do not contain the C2 domain, and C2CD4D contains a shorter C2 domain.

A previous study suggested that C2CD4A and C2CD4B are localized in the nucleus in COS‐7 cells 18. We examined the intracellular localization of C2CD4C, by overexpressing Flag‐tagged or HA‐tagged C2CD4C in MIN6 and COS‐7 cells. With both N‐terminal and C‐terminal tags, C2CD4C was found to be localized in the cytoplasm, but not in the nuclei, of both cell types (Fig. 1D). These results suggested that C2CD4C functions might differ from those of C2CD4CA and C2CD4B. This encouraged us to study the role of *C2cd4c*.

### Generation of *C2cd4c/LacZ* KI mice

**Figure 2:**
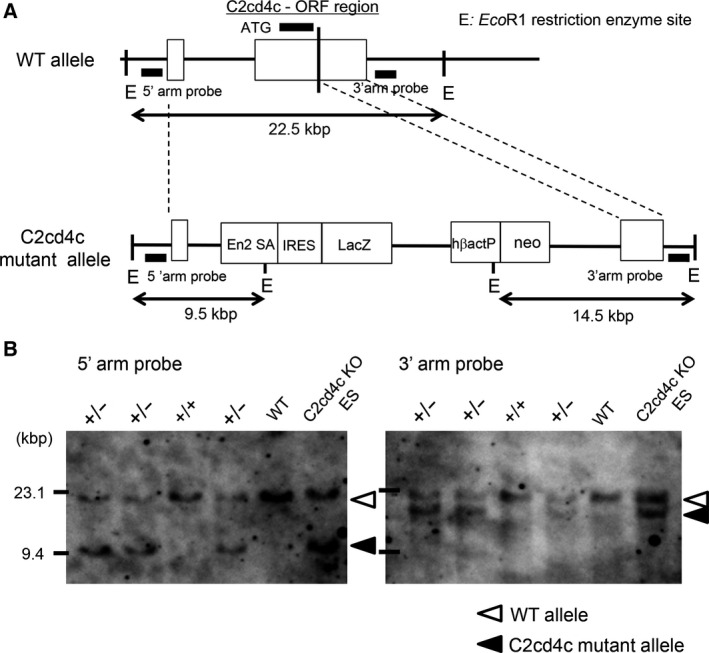
Generation of *C2cd4C*/*LacZ* knock‐in (KI) mice. (A) The *LacZ* gene was inserted into the *C2cd4c* locus. The genome of *C2cd4c*/*LacZ* KI mice was verified by *Eco* RI restriction enzyme digestion and Southern blot analysis using DNA probes in the 5′‐arm or 3′‐arm. +/‐: heterozygous KI mice; WT: wild‐type mice; C2cd4c KI ES: KI ES cells from which the mice were derived. (B) Southern blot analysis of DNA from *C2cd4c*/*LacZ* KI mice.

To generate *C2cd4c*/*LacZ* KI mice, we obtained the JM8A3‐A10 embryonic stem (ES) cell line from the KOMP Repository 12, 13, 14. JM8A3‐A10 ES cells with the *LacZ* gene inserted into the ORF of the *C2cd4c* locus were generated by homologues recombination (Fig. 2A). *C2cd4c*/*LacZ* KI ES cells were then injected into the blastocysts of C57/BL6 mice to generate *C2cd4c*/*LacZ* KI mice. To confirm the correct insertion of the *LacZ* gene into the *C2cd4c* locus, genomic DNA of the mutant or control wild‐type mice was digested using *Eco*R1 restriction enzyme and processed for Southern blot analysis, using the 5′‐ or 3′‐arm as the probes. In mutant mice, the 5′‐probe and the 3′‐probe detected products of 9.5 or 14.5 kb, respectively. In contrast, in wild‐type mice, both probes detected products of 22.5 kb (Fig. 2A,B). From these results, we confirmed the generation of *C2cd4c*/*LacZ* KI mice.

### C2cd4c is expressed in the developing and in the adult pancreas

**Figure 3:**
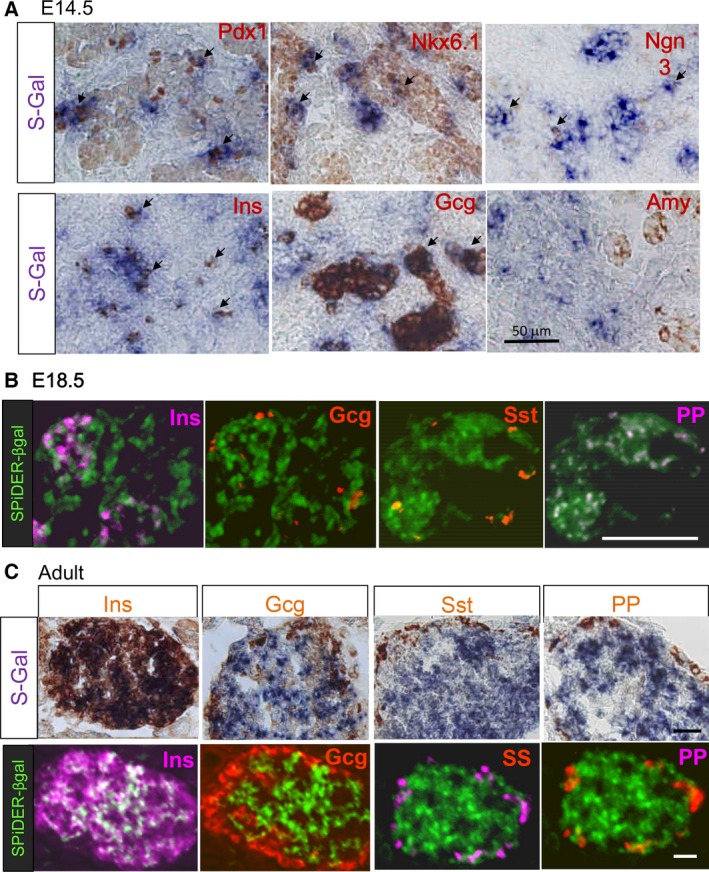
C2cd4C is expressed in embryonic endocrine cells and in pancreatic beta cells. Immunohistochemical analysis of the developing pancreas and adult islets by S‐gal or SPiDER‐βGal staining. (A) Immunohistochemical analysis of the pancreas at E14.5. LacZ staining was observed in Pdx1+, Nkx6.1+, Ngn3+, Insulin (Ins)+, and Glucagon (Gcg)+ positive endocrine cells but not in Amylase (Amy)+ exocrine cells. Arrows depict cells showing overlapping stainings. (B) Immunohistochemical analysis of the pancreas at E18.5. Many Insulin+ cells and pancreatic polypeptide (PP)+ cells were positively stained for LacZ. Few Glucagon+, Somatostatin (Sst)+ cells were positively stained for LacZ. (C) Immunohistochemical analysis and LacZ staining of adult islets, using S‐Gal or SPiDER‐βGal as substrates. Insulin+ cells were positively stained for LacZ, but other endocrine cells were negative. Scale bar; 100 μm.

We then examined the expression of *C2cd4c* in the pancreas by visualizing *LacZ* activity. For this, we used Salmon‐gal (S‐gal; 6‐chloro‐3‐indolyl‐beta‐d‐galactopyranoside) in combination with Nitro Blue tetrazolium 15, or SPiDER‐βGal, which is rendered fluorescent by the enzymatic reaction 16. We visualized *C2cd4c*/*LacZ* activity during embryonic development (at E14.5–18.5) and in adult *C2cd4c*/*LacZ* KI heterozygous mice (Fig. 3). To clarify the localization of LacZ staining in the pancreas, we performed immunohistochemical analysis of pancreatic markers after S‐gal or SPiDER‐βGal staining (Fig. 3). At E14.5, LacZ activity visualized by S‐gal staining was observed in Pdx1‐expressing pancreatic epithelium. Particularly, LacZ staining overlapped with Pdx1‐strong positive cells in the trunk region, which are known to give rise to the endocrine cells 5. We stained with the endocrine progenitor markers Nkx6.1 and Ngn3 8, 9 and found that some LacZ‐positive cells also expressed Nkx6.1 or Ngn3. The regions of LacZ‐positive cells were smaller than those of Pdx1‐ or Nkx6.1‐expressing cells. However, Ngn3‐expressing cells are fewer in number and seem to lie within the LacZ‐positive region. Costaining for insulin, glucagon, or amylase revealed that the LacZ‐positive cells overlapped with insulin‐ or glucagon‐expressing endocrine cells, but not with the amylase‐expressing acinar cells 3. At E18.5, many SPiDER‐βGal‐positive cells overlapped with insulin‐ or pancreatic polypeptide‐positive cells, but few overlapped with glucagon‐ or somatostatin‐positive cells (Fig. 3B). We confirmed that S‐Gal staining (Fig. 3C, upper panels) and SPiDER‐βGal staining (Fig. 3C, lower panels) gave similar results in the adult islets. In the adults, LacZ‐positive cells expressed insulin but none of the other endocrine markers (Fig. 3C). Taken together, these results indicate that *C2cd4c* is expressed in early endocrine progenitors during the embryonic stages, then gradually localized to insulin‐expressing β cells and PP cells, then solely in the insulin‐expressing β cells in the islets of the adult pancreas.

### C2cd4c is dispensable for pancreatic development

**Figure 4:**
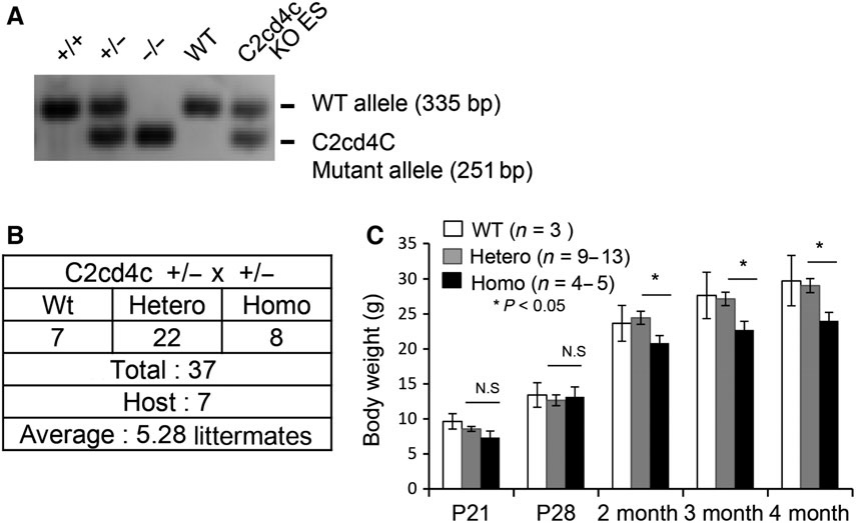
Establishment of *C2cd4c* knockout (KO) mice. (A, B) Establishment of *C2cd4c* gene KO mice. (A) Genomic PCR analysis of *C2cd4c* KO mice. (B) *C2cd4c* KO mice were born in agreement with a Mendelian distribution (Chi‐square test, *P* = 0.5). (C) Measurement of body weight. The body weights of KO homozygous mice were significantly decreased compared to those of wild‐type and heterozygous mice.

**Figure 5:**
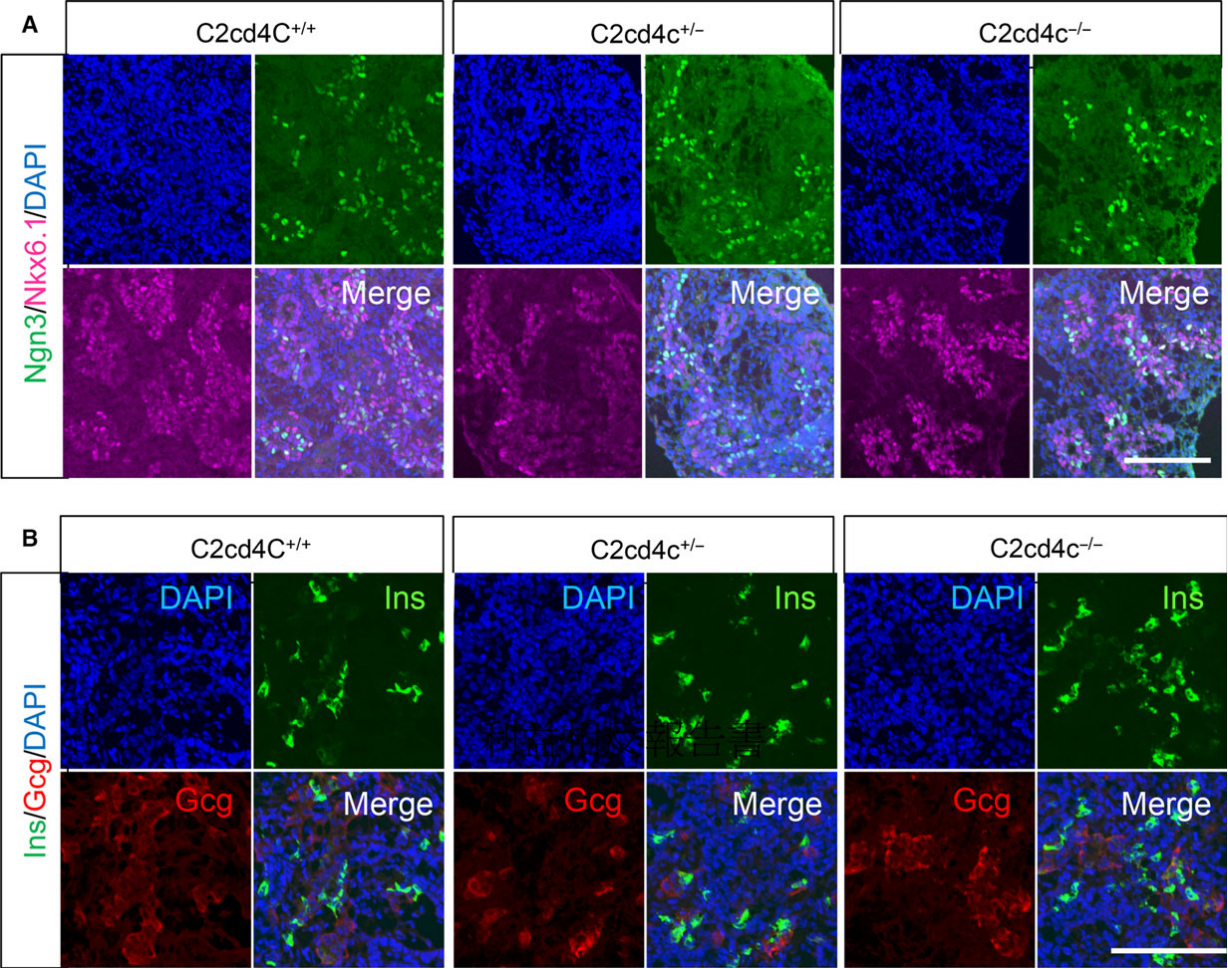
Endocrine cells are normal in *C2cd4c* mutant mice at E 14.5. Immunohistochemical analysis of Ngn3 and Nkx6.1 (A), Insulin and Glucagon (B) at E 14.5 in the pancreas of wild‐type (C2cd4c^+/+^), heterozygous mutant (C2cd4c^+/−^), and KO (C2cd4c^−/−^) mice. Scale bar; 100 μm.

**Figure 6:**
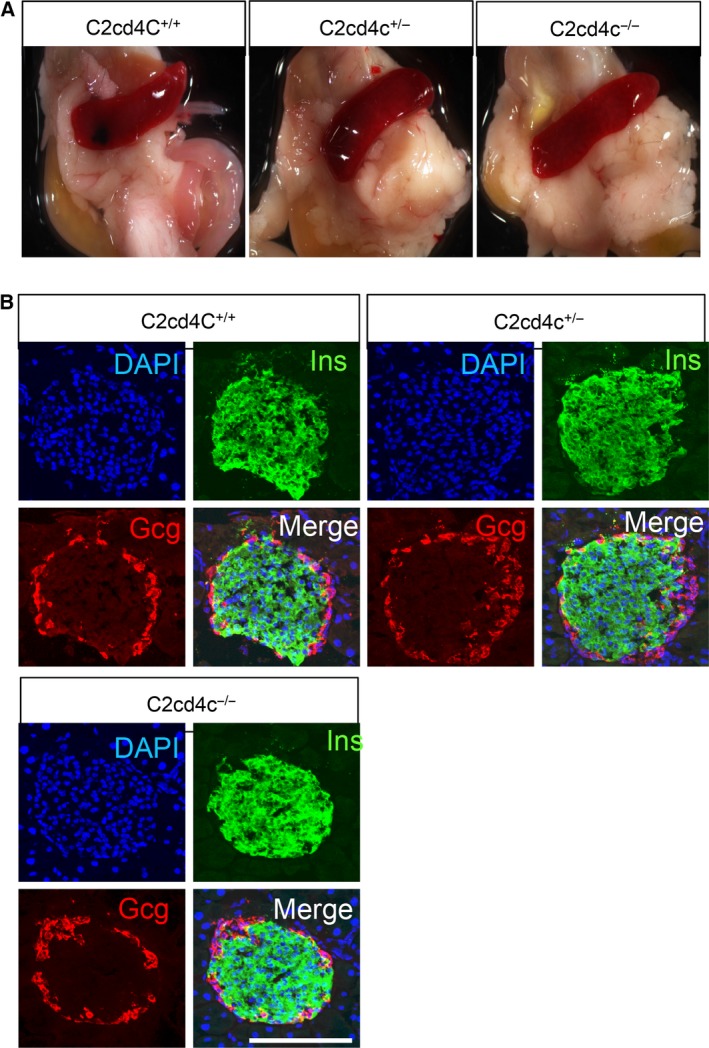
Pancreatic islets develop normally in *C2cd4c* mutant mice. (A) The morphology of the pancreas in KO mice with wild‐type and heterozygous KO mice as controls. (B) Immunohistochemical analysis of Insulin and Glucagon expression in islets of KO, heterozygous KO, and wild‐type mice. Scale bar; 100 μm.

**Figure 7:**
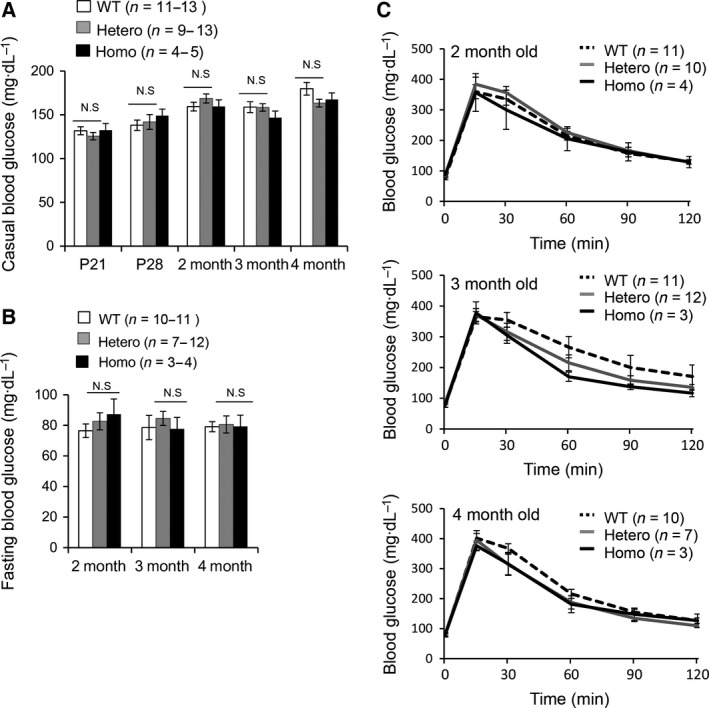
*C2cd4c* mutant mice show normal blood glucose levels. (A) Nonfasted blood glucose, (B) fasting blood glucose, and (C) glucose tolerance were normal in *C2cd4c* mutant mice (Homo) as compared to wild‐type (WT) and heterozygous (Hetero) mutant mice at the indicated ages.

We then analyzed *C2cd4c* KO (−/−) mice (Fig. 4A). *C2cd4c* KO mice were born following Mendelian distribution (Fig. 4B). The body weight of KO mice was slightly lower than that of *C2cd4c* +/− heterozygous mice at 2 months after birth (Fig. 4C). There seems no marked differences in food intake (H. Omori, unpublished). As *C2cd4c* was expressed in the endocrine region of the embryonic pancreas and in β cells of the adult, we examined pancreatic development of KO mice. At E14.5, the endocrine progenitor markers Ngn3 and Nkx6.1 were normally expressed in KO mice (Fig. 5A), and embryonic alpha or beta cells identified by glucagon or insulin expression were formed normally (Fig. 5B). Amylase‐expressing acinar cells and *Dolichos biflorus* Agglutinin (DBA)‐expressing ductal cells were also normal in the KO mice (Fig. S1). We then examined the adult pancreas of KO mice. KO mice showed normal pancreas and islet morphology (Fig. 6A,B). We tested the pancreatic function of KO mice, because *C2cd4c* is expressed in β cells that function to maintain blood glucose homeostasis. Both nonfasted and fasting blood glucose levels in KO mice were normal (Fig. 7A,B). Intraperitoneal glucose tolerance test (IPGTT) also revealed that KO mice showed normal blood glucose tolerance after glucose challenge (Fig. 7A). We confirmed by semiquantitative RT‐PCR analysis that no marked overexpression of *C2cd4a* or *C2cd4b* was observed in the *C2cd4c* KO mice (Fig. S2). These results therefore suggest that C2cd4c is dispensable for normal pancreatic development.

## Discussion

Several groups have used comprehensive genome wide analysis to identify genes that play roles in the proliferation and differentiation of pancreatic beta cells 19. Previously, we reported that *C2cd4b* is expressed in the embryonic pancreas 4. Here, we focused on *C2cd4c,* which is well conserved across species and contains the C2 domain with the Ca^2+^‐binding motif 20. C2 domains are independently folded modules of about 130 residues, and form a compact β sandwich of two 4‐stranded β‐sheets 11, 21. Many of the C2 proteins are involved in membrane trafficking and fusion, and serve as Ca^2+^ effectors for divergent Ca^2+^‐mediated cellular processes 22. The C2 domain‐containing proteins such as synaptotagmin7, double C2‐like domain‐containing proteins alpha and beta (Doc2α and Doc2β), and C2 domain‐containing transmembrane protein 24 (Tmem24), are thought to bind to Ca2+ and function as Ca2+ sensors for the rapid phase of release in synaptic transmission or insulin exocytosis 21, 23, 24, 25. In the pancreas, C2CD4C is the only C2CD4 family member that contains a C2 domain and is expressed from developing stages and in the adult islets. This suggests that *C2cd4c* might have some functions related to insulin exocytosis, which encouraged us to focus on *C2cd4c*.

*C2cd4c* is strongly expressed in the trunk region of the E14.5 embryonic pancreas, where endocrine progenitor cells are located, and in the adult islets. The period between E12.5 and E15.5 is known as the second transition of the embryonic pancreas, during which differentiation into the endocrine and exocrine cells is observed 7; Nkx6.1 and Ngn3 are expressed in the trunk region 1, 9. We identified that *C2cd4c* expression overlapped with that of these endocrine progenitor markers. At E18.5, *C2cd4c* was expressed in the insulin‐expressing cells and in the pancreatic polypeptide‐expressing cells, although only a few glucagon‐expressing cells and somatostatin‐expressing cells seemed to express *C2cd4c*. These results suggest that *C2cd4c* is gradually confined to the beta cells of the embryonic pancreas, and then becomes restricted to β cells in the adult.

*C2cd4c* KO mice were born following Mendelian distribution and were healthy. Fasting blood glucose levels and glucose tolerance were normal. However, the weight of KO mice was significantly less than that of heterozygous mice, which however seemed not due to less food intake by visual inspection. The reason for the lower body weight in the homozygous mice remains to be determined. Although *C2cd4c* is expressed during pancreatic development, the expression of pancreatic endocrine genes *Ngn3*,* Nkx6.1*, insulin, and glucagon was not affected in KO mice. Pancreatic islets also developed normally. Our results indicate that C2cd4c is not required for normal pancreatic development. However, *C2cd4c* might play a role in β cell regeneration and might show redundant roles with other members of the *C2cd4* family, which remains to be investigated. Recently, Synaptagmin 4, lacking a C2 domain, was reported to have an inhibitory role for exocytotic activity 26. Therefore, it might be useful to investigate further into the function of *C2cd4c* with respect to the other members of the *C2cd4* family genes in the regulation of insulin exocytosis.

## Author contribution

HO contributed to the acquisition and analysis of data and drafting manuscript. SO was responsible for concept and design, acquisition of data; analysis and interpretation of data and drafting manuscript. DS and MS were responsible for acquisition and analysis of data. KU and DS discussed the data, provided technical advice. NT performed blastocyst injection of the ES cells and generated gene KI mice. NN provided technical advice and support for the maintenance of gene KO mice. SK provided conceptual input, discussion, writing, and revision of the manuscript; approved the final version of the manuscript and obtained funding.

## Supporting information


**Fig. S1.** Acinar and duct cells are not affected in the knockout (KO) mice.

**Fig. S2.** No marked increase in *C2cd4a* or *C2cd4b* expression in *C2cd4c* KO mice.

**Table S1.** Primers used to detect gene expressions.
