# Supplementary figures and images for: Changes in expression of C2cd4c in pancreatic endocrine cells during pancreatic development
Source: FEBS Lett. 2016 Jul 14;590(16):2584–93. doi: 10.1002/1873-3468.12271 (PMC5129588; doi:10.1002/1873-3468.12271)

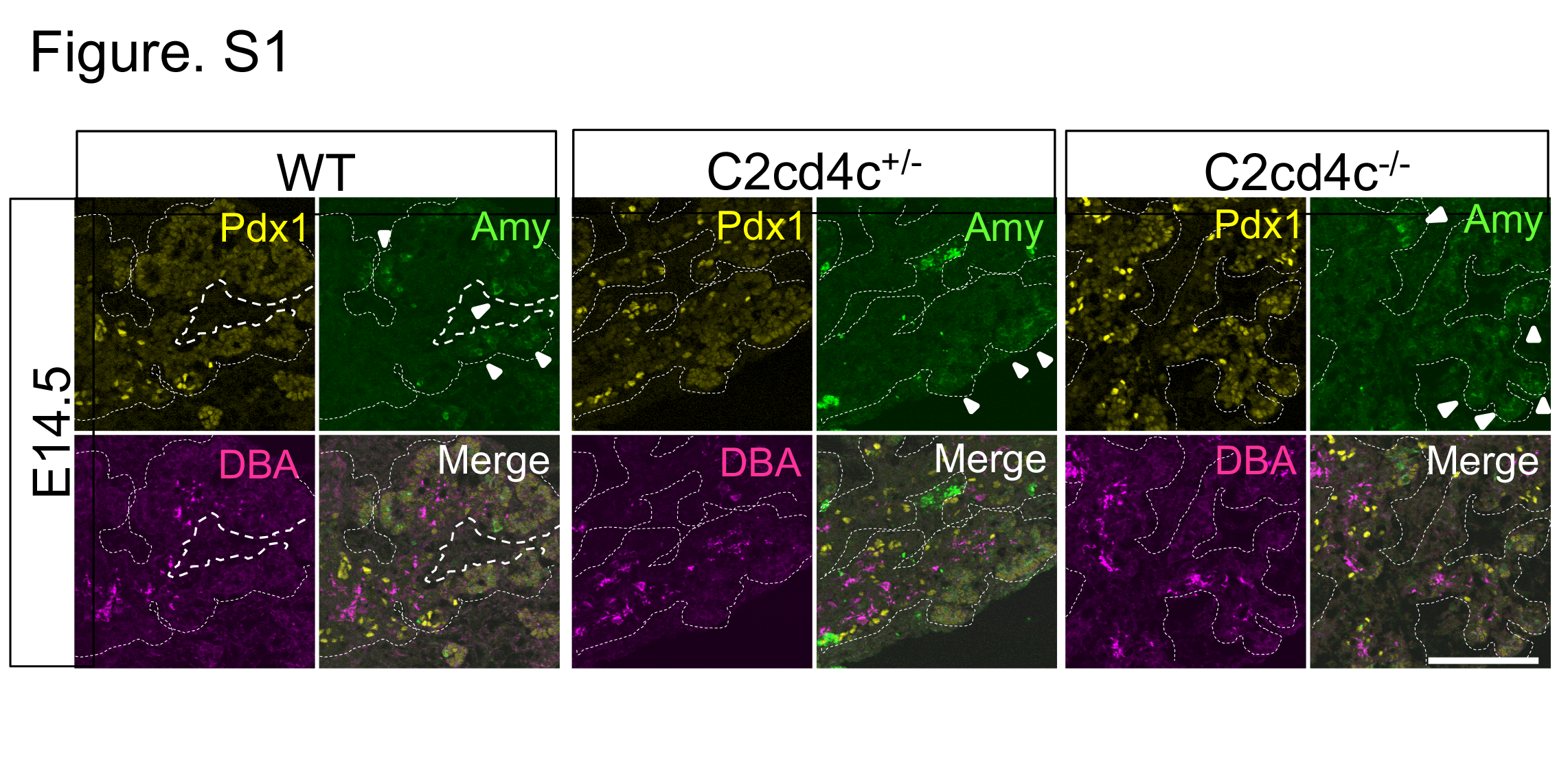

Supplement: Supplementary file 1 — Fig. S1. Acinar and duct cells are not affected in the knockout (KO) mice. [file FEB2-590-2584-s001.tif]

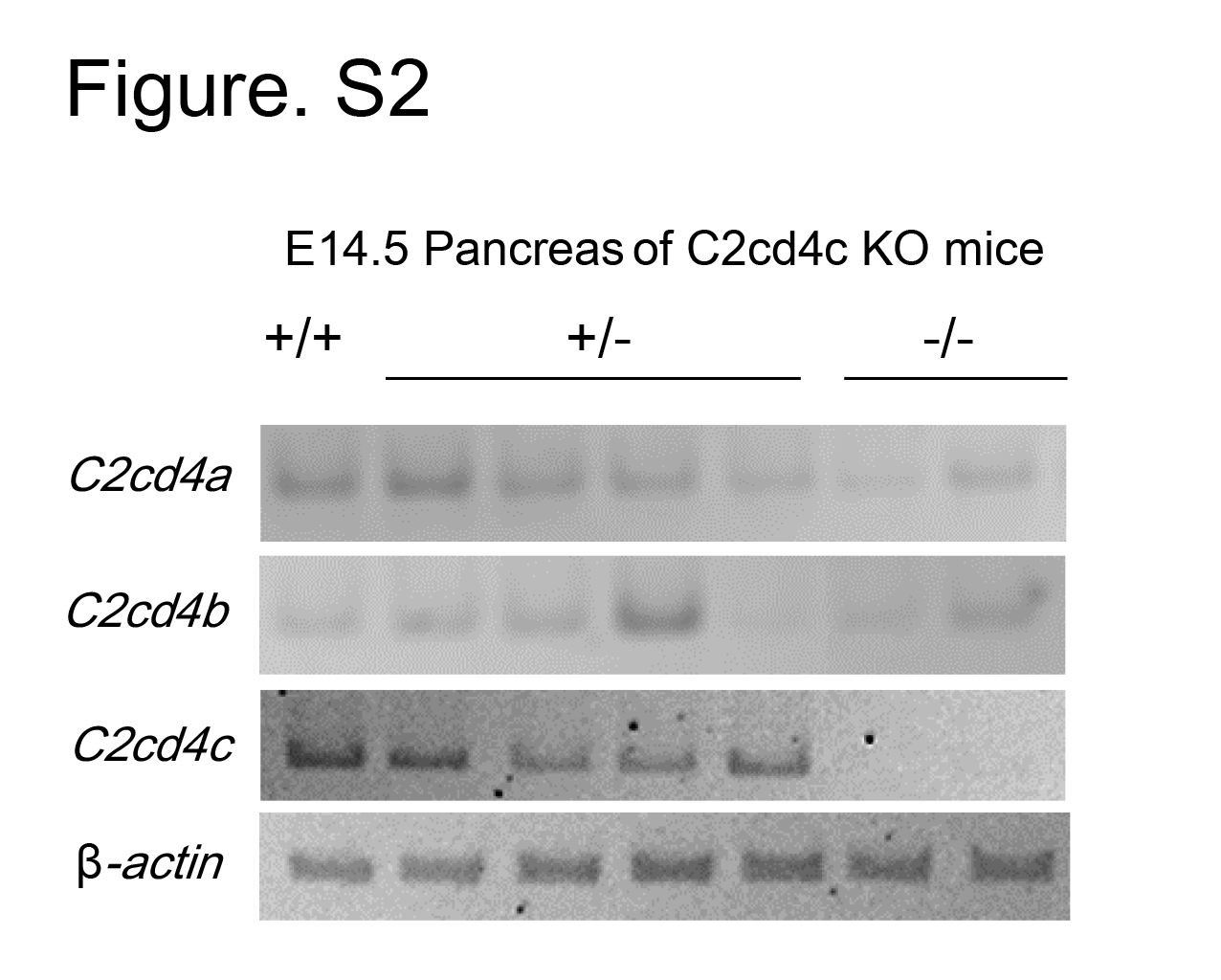

Supplement: Supplementary file 2 — Fig. S2. No marked increase in C2cd4a or C2cd4b expression in C2cd4c KO mice. [file FEB2-590-2584-s002.tif]

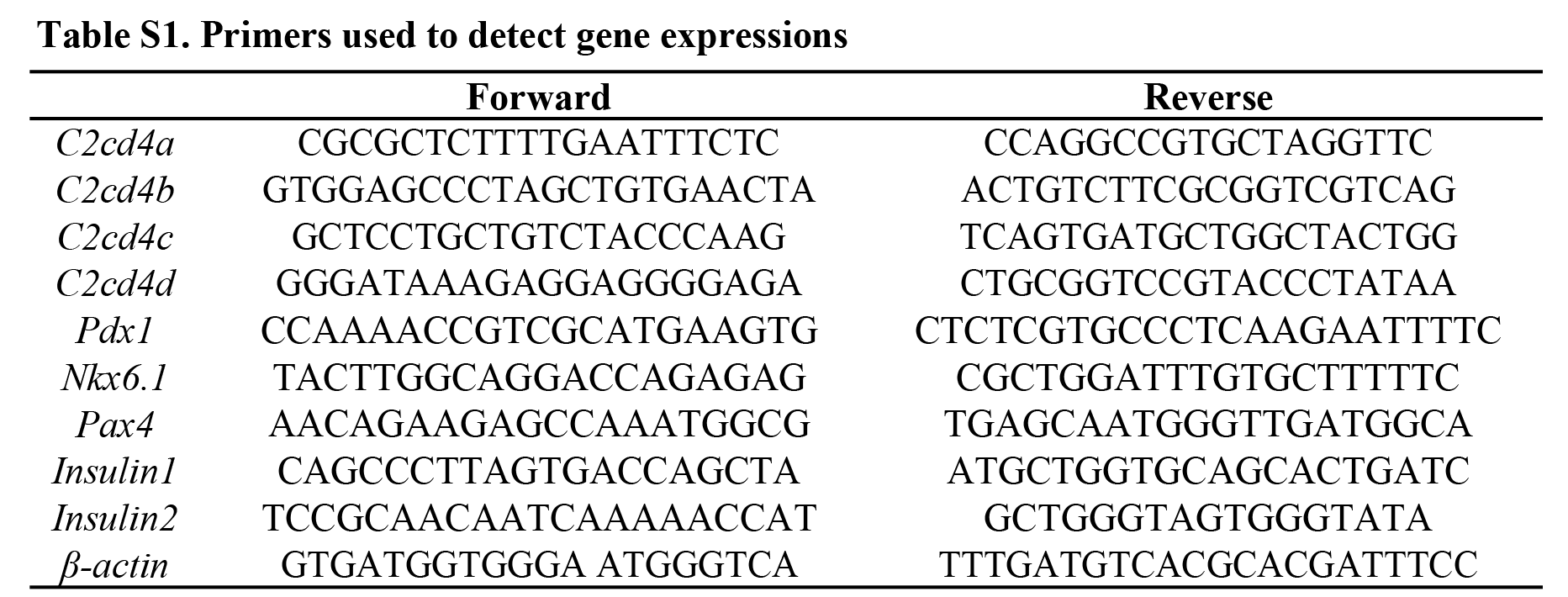

Supplement: Supplementary file 3 — Table S1. Primers used to detect gene expressions. [file FEB2-590-2584-s003.tif]
